# Pair of lice lost or parasites regained: the evolutionary history of anthropoid primate lice

**DOI:** 10.1186/1741-7007-5-7

**Published:** 2007-03-07

**Authors:** David L Reed, Jessica E Light, Julie M Allen, Jeremy J Kirchman

**Affiliations:** 1 Florida Museum of Natural History, University of Florida, Gainesville, Florida 32611, USA; 2 Department of Zoology, University of Florida, Gainesville, Florida 32611, USA; 3 New York State Museum, 3140 CEC, Albany, NY 12230, USA

## Abstract

**Background:**

The parasitic sucking lice of primates are known to have undergone at least 25 million years of coevolution with their hosts. For example, chimpanzee lice and human head/body lice last shared a common ancestor roughly six million years ago, a divergence that is contemporaneous with their hosts. In an assemblage where lice are often highly host specific, humans host two different genera of lice, one that is shared with chimpanzees and another that is shared with gorillas. In this study, we reconstruct the evolutionary history of primate lice and infer the historical events that explain the current distribution of these lice on their primate hosts.

**Results:**

Phylogenetic and cophylogenetic analyses suggest that the louse genera *Pediculus *and *Pthirus *are each monophyletic, and are sister taxa to one another. The age of the most recent common ancestor of the two *Pediculus *species studied matches the age predicted by host divergence (ca. 6 million years), whereas the age of the ancestor of *Pthirus *does not. The two species of *Pthirus *(*Pthirus gorillae *and *Pthirus pubis*) last shared a common ancestor ca. 3–4 million years ago, which is considerably younger than the divergence between their hosts (gorillas and humans, respectively), of approximately 7 million years ago.

**Conclusion:**

Reconciliation analysis determines that there are two alternative explanations that account for the current distribution of anthropoid primate lice. The more parsimonious of the two solutions suggests that a *Pthirus *species switched from gorillas to humans. This analysis assumes that the divergence between *Pediculus *and *Pthirus *was contemporaneous with the split (i.e., a node of cospeciation) between gorillas and the lineage leading to chimpanzees and humans. Divergence date estimates, however, show that the nodes in the host and parasite trees are not contemporaneous. Rather, the shared coevolutionary history of the anthropoid primates and their lice contains a mixture of evolutionary events including cospeciation, parasite duplication, parasite extinction, and host switching. Based on these data, the coevolutionary history of primates and their lice has been anything but parsimonious.

## Background

Sucking lice (Phthiraptera: Anoplura) are permanent and obligate ectoparasites of eutherian mammals. These highly specialized blood-sucking insects live in close association with their hosts and complete their entire life cycle on the host [1]. Anoplurans have modified mouthparts for feeding on host blood and because mammalian blood differs widely among species in terms of its suitability for louse nutrition [2], sucking lice can be highly host specific [1,3]. Host specificity could also be reinforced by interactions with the host's immune system. High host specificity can arise from a long history of cospeciation between hosts and their parasites. Cospeciation is speciation (or cladogenesis) in a parasite lineage as a result of, or at the same time as, host cladogenesis [4]. The current distribution of parasites on host taxa (the host-parasite associations) can be the result of cospeciation or various historical events [5] such as host switching, sorting events (extinction and lineage sorting), duplication events (parasites speciating on a single host lineage), and failure of the parasite to speciate when the host speciates ('missing the boat'; [6]). These historical processes can be detected by comparing the phylogenies of hosts and their parasites using methodologies such as reconciliation analysis [7]. Previous cophylogenetic studies of lice (both sucking lice and chewing lice) have documented each of these historical events in various combinations (for a review, see [8]).

**Figure 1:**
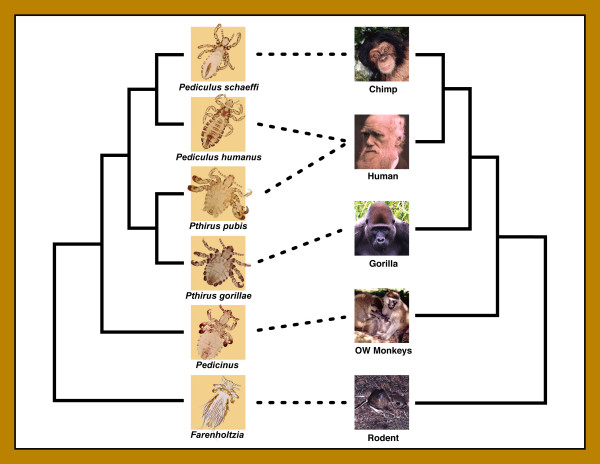
**Phylogenetic trees for primate lice and their vertebrate hosts redrawn from Reed *et al*. [9]**. Trees are shown as cladograms with no branch length information, and are based on molecular and morphological data. Dashed lines between trees represent host-parasite associations. Humans are unique in being parasitized by two genera (*Pediculus *and *Pthirus*). Photo credits: J. W. Demastes, T. Choe, and V. Smith.

Humans (*Homo sapiens*) are parasitized by two genera of sucking lice: one shared with chimpanzees (*Pan *spp.) and the other shared with gorillas (*Gorilla gorilla*). Human head and body lice, as well as chimpanzee lice, are members of the genus *Pediculus *(*Pediculus humanus *and *Pediculus schaeffi*, respectively). There is no *Pediculus *species known to parasitize gorillas. Human pubic lice and gorilla lice belong to the genus *Pthirus *(*Pthirus pubis *and *Pthirus gorillae*, respectively), and no *Pthirus *species is known to parasitize chimpanzees. *Pediculus *and *Pthirus *are sister taxa based on morphology and molecular data (Figure 1), and primate lice are known to have cospeciated with their hosts for at least 25 million years [9]. The curious distribution of these two genera raises an interesting question regarding the evolutionary history of primate lice. Why do humans retain both genera, but chimpanzees and gorillas have only one genus each?

**Figure 2:**
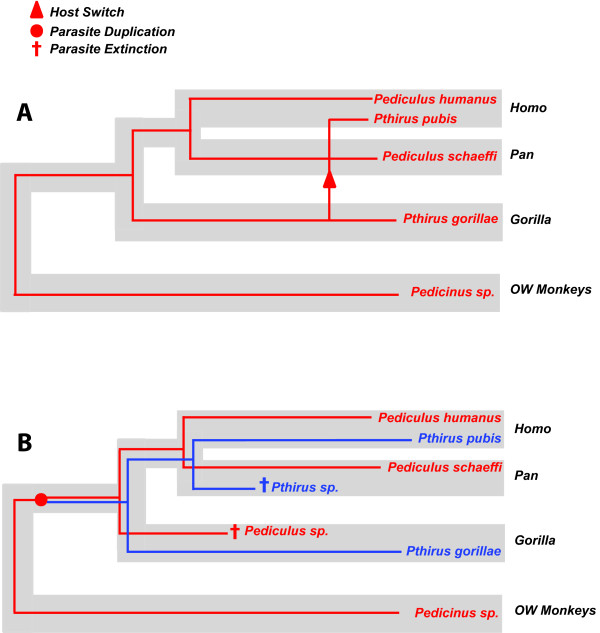
**Cophylogenetic reconstructions with the host phylogeny for humans, chimpanzees, gorillas, and Old World monkeys indicated by thick grey lines, and the louse phylogeny indicated by thin red and blue lines**. (A) Reconstruction showing perfect cospeciation between with hosts and parasites with the exception of a single host switch of *Pthirus sp*. from gorillas to humans (marked by an arrow). (B) Cophylogenetic reconstruction showing an ancient duplication creating two evolutionarily distinct lineages (*Pediculus *and *Pthirus*), each having cospeciated with gorillas, chimps, and humans with two extinction events (marked with daggers). The reconciliation shown in panel A requires one evolutionary step (the host switch), whereas reconciliation B requires three steps (one duplication and two extinctions).

Given what is already known about the coevolutionary history of the lice and their hosts, we can speculate that there are two mutually exclusive explanations that can account for the current distribution of *Pediculus *and *Pthirus *(Figure 2). The most parsimonious explanation (i.e., the explanation requiring the fewest number of steps) predicts perfect cospeciation between the primates and lice with the addition of a single host switch. In this scenario, the divergence between *Pediculus humanus *and *Pediculus schaeffi *occurred at the same time as the split between their hosts, humans and chimpanzees, ca. 6 million years ago (MYA; [10]), and the split between *Pediculus *and *Pthirus *occurred contemporaneously with the split between gorillas and the lineage leading to chimpanzees and humans (ca. 7 MYA; Figure 2A; [10]). These events were followed some time later by one host switch of a *Pthirus *species from gorillas to humans (Figure 2A). Host switching among lice is common in many groups of birds and mammals [11-13]. This 'recent host switch' hypothesis requires one evolutionary step and predicts that the divergence between *Pthirus pubis *and *Pthirus gorillae *is more recent than the chimpanzee/human split (Figure 2A). How we might have acquired our pubic lice from gorillas is not immediately apparent, however it would be interesting to know whether the switch was very recent (say less than 100,000 years old) or whether it was considerably older.

The alternative hypothesis involves an ancient louse duplication event that occurred on the ancestor of gorillas, chimpanzees, and humans, which would have created the lineages leading to the two extant genera, *Pediculus *and *Pthirus *(Figure 2B). In this case, the timing of the divergence between *Pthirus pubis *and *Pthirus gorillae *would correspond to that of their hosts (ca. 7 MYA; [10]). In this scenario, humans would have retained both genera, but chimpanzees would have lost a *Pthirus *species and gorillas would have lost a *Pediculus *species to extinction (Figure 2B). Such parasite duplications and extinctions are common in the lice of birds and mammals (e.g., [14]). This parasite extinction or 'pair of lice lost' model is less parsimonious than the 'recent host switch' hypothesis listed above because it requires at least three evolutionary steps (a duplication of the parasite on a primate common ancestor as well as the extinction of one *Pediculus *and one *Pthirus *lineage, Figure 2B). Although the 'recent host switch' hypothesis is more parsimonious, it is not necessarily more likely than the 'pair of lice lost' hypothesis. Each of the historical events (host switch, duplication, and extinction) has some probability of occurrence, the quantification of which is beyond the scope of this paper. There are additional hypotheses that can be postulated that have more evolutionary steps than the two presented above, however there are no other hypotheses that have an equal number of steps or fewer steps. For instance, one might assume that the current distribution of lice resulted from a host switch of *Pthirus *from humans to gorillas (the opposite direction of the switch in the 'recent host switch' model). However, this evolutionary scenario would require at least five evolutionary steps.

The two hypotheses of parasite distributions ('pair of lice lost' and 'recent host switch') are based on the premise of maximizing the number of cospeciation events and minimizing the number of events that deviate from cospeciation, which are typical of analyses that attempt to reconcile host and parasite associations that are based on the concept of evolutionary parsimony (reconciliation analysis as implemented in TreeMap; [7]). However, parsimony-based reconciliation analyses do not take into account branch lengths and divergence times – information that is essential to distinguish between the 'recent host switch' and 'pair of lice lost' hypotheses. In the presence of significant cospeciation we can use the timing of speciation events to differentiate among alternative hypotheses of host-parasite associations [15].

We have performed phylogenetic and cophylogenetic analyses of two genes, the mitochondrial cytochrome *c *oxidase subunit I (Cox1) gene and nuclear gene elongation factor 1 alpha (EF-1α) gene, to determine the shared evolutionary history of primate lice and their hosts. We also investigate the use of standard phylogenetic methods for reconstructing coevolutionary histories when standard cophylogenetic methods (e.g., reconciliation analysis) cannot always find the solution that best fits the observed data.

## Results

### Phylogenetic and cophylogenetic analyses

**Figure 3:**
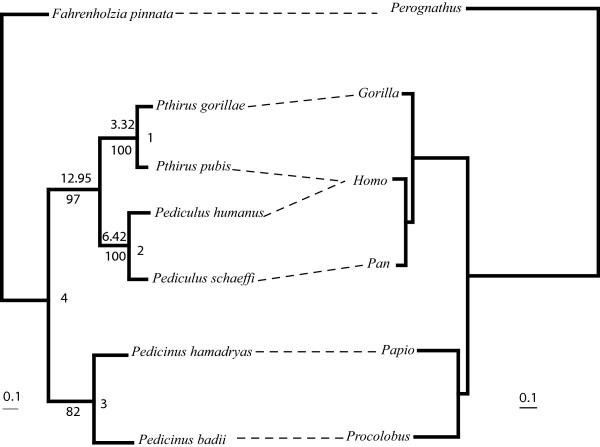
**Maximum likelihood (ML) phylogeny of primate lice using the combined Cox1 and EF-1α dataset with a best-fit ML model of nucleotide substitution (left)**. Bootstrap values are indicated below the nodes and divergence estimates are given above. Clade numbers used in Table 1 are provided to the right of each node. The ML phylogeny of the Cox1 gene from host taxa is indicated on the right. Branch lengths are drawn to the same scale (substitutions/site), and are based on the best-fit ML model of nucleotide substitution. Dashed lines connect hosts and their associated parasites.

The partition homogeneity test determined that the Cox1 and EF-1α genes did not differ significantly (*p *= 0.94), therefore a combined analysis was performed in addition to analyses based on single genes. The best-fit model for each individual gene and the combined-gene analysis of lice was a transversion model (TrN+I+Γ model) that permitted two nucleotide substitution rates for transversions, one rate for transitions, unequal base frequencies, a rate heterogeneity parameter (G), and a parameter for invariant sites (I). Similarly, the best-fit model for the Cox1 gene from the primate host taxa was also TrN+I+Γ. Maximum likelihood (ML) analysis of the combined Cox1 and EF-1α dataset (as well as individual gene datasets) from lice produced a single, well-supported phylogeny in agreement with results from previous analyses (Figure 3; [9]). When the same ML analysis was performed enforcing a molecular clock, the resulting tree topology did not change and the resulting tree score was not significantly different than the unconstrained score (*p *= 0.7625). The ML analysis of the host Cox1 data also resulted in a single phylogeny in agreement with known relationships among these primate taxa (Figure 3).

Cophylogenetic analyses using TreeMap [7] produced two reconciliations of host and parasite phylogenies in agreement with the 'recent host switch' and 'pair of lice lost' hypotheses presented in Figure 2. The reconciliation concordant with the 'recent host switch' hypothesis (Figure 2A) included five cospeciation events and one host switch for a total cost of 1.0. The reconstruction concordant with the 'pair of lice lost' hypothesis (Figure 2B) was less parsimonious. While this reconstruction included five cospeciation events, there was also a single duplication event and two losses resulting in a total cost of 3.0. Both reconciliations show significantly greater similarity between the host and parasite trees than would be expected based on chance alone (i.e., both reconciliations show significant cospeciation, *p *< 0.05).

### Divergence date estimation

Divergence date estimates differed little between r8s and multidivtime analyses and between individual and combined genes (Table 1). For convenience we will refer to divergence date estimates based on the combined gene tree used in *multidivtime *(Table 1 and Figure 3). Mean divergence date estimates for the split between the chimpanzee and human head/body lice (*Pediculus schaeffi *and *Pediculus humanus*, respectively) averaged 6.39 MYA. The divergence date estimates for the gorilla and human pubic lice (*Pthirus gorillae *and *Pthirus pubis*, respectively) averaged 3.32 MYA and are noticeably more recent than the split between the two *Pediculus *species. The estimated divergence date for the most recent common ancestor (MRCA) of the two genera, *Pthirus *and *Pediculus*, was estimated to be 12.95 MYA (Table 1 and Figure 3), noticeably older than the MRCA of chimpanzees, humans, and gorillas.

## Discussion

Reconciliation analysis using TreeMap corroborates earlier reports of significant cospeciation between primate lice and their hosts [9]. These cophylogenetic analyses also result in two reconciliations of the host and parasite phylogenies where the most parsimonious reconstruction favors the 'recent host switch' hypothesis (Figure 2A). However, divergence date estimates conflict with the results of the reconciliation analysis because the 'recent host switch' hypothesis predicts that the divergence of *Pediculus *and *Pthirus *would be roughly contemporaneous with the split between gorillas and the lineage leading to humans and chimpanzees. Our estimates of the MRCA of *Pediculus *and *Pthirus *dates to roughly 13 MYA, not remotely consistent with the MRCA of humans, chimpanzees, and gorillas (ca. 7 MYA; [10]). Given the much older age of our MRCA of *Pediculus *and *Pthirus*, it is more appropriate, although less parsimonious, to assume that the origin of the two genera was the result of a parasite duplication event rather than a cospeciation event ('pair of lice lost' hypothesis; Figure 2B). It is curious that the estimate of the MRCA of *Pthirus *and *Pediculus *(13 MYA) is contemporaneous with the divergence of Orangutans from other apes [10], however this is possibly coincidental. Lice do not parasitize orangutans; therefore, reconstructing their role in the evolutionary history of primate sucking lice will be difficult.

The 'pair of lice lost' hypothesis is also unsatisfactory when compared to divergence date estimates. For the 'pair of lice lost' hypothesis to be correct we must assume that divergence between *Pthirus pubis *and *Pthirus gorillae *is roughly contemporaneous with the split of gorilla from the lineage leading to chimpanzees and humans (i.e., ca. 7 MYA). Our divergence date estimates of roughly 3–4 MYA (Table 1) is much younger than the host divergence of 7 MYA [10] and is even younger than the divergence between chimpanzees and humans (ca. 6 MYA).

The estimates of divergence dates argue for a more complex evolutionary history than estimated by reconciliation analysis. While reconciliation analysis serves to find the most parsimonious reconstruction of host and parasite evolutionary history by maximizing cospeciation events and minimizing the cost of the reconstruction, it can only identify possible scenarios describing the evolutionary history between associated taxa. Incorporating branch length data in other analyses is necessary to determine which scenario best fits the observed data. For example, post-hoc Mantel tests are commonly used to look for overall correlation in host and parasite data sets [16]. However, we have too few taxa to perform such an analysis, and we have instead relied upon ad-hoc phylogenetic tests to determine whether certain nodes of cospeciation were contemporaneous.

To further examine the validity of the 'pair of lice lost' hypothesis, we assessed whether the divergence of the two *Pthirus *species was contemporaneous with the host divergence of roughly 7 MYA. The branch length between the two *Pthirus *species in the best-fit ML louse tree was artificially lengthened to approximate a branch that is the same age as the branch between *Pediculus schaeffi *and *Pediculus humanus*, thereby representing the split between chimp and human lice. It is widely known that the gorilla, chimpanzees, and human divergence times are very close in age, so if our estimate is contemporaneous with the divergence of chimpanzees and humans (the most conservative expected age), then we cannot rule out true contemporaneous times of divergence between the two *Pthirus *species and their hosts. The likelihood score of this constrained tree (with a branch length approximating a 6 million year divergence between *Pthirus gorillae *and *Pthirus pubis*) was significantly worse than the true louse tree (where the estimated divergence was closer to 3–4 MYA; d.f. = 6, χ^2 ^= 12.91, *p *= 0.044). We can therefore reject contemporaneous divergence events and we can conclude that the split between the human and gorilla species of *Pthirus *diverged much more recently than the split between humans and gorillas or even humans and chimpanzees. The divergence within *Pthirus *is therefore the result of a host switch from gorillas to humans (loosely defined) roughly 3–4 MYA, as predicted in the 'recent host switch' hypothesis.

We can similarly examine the divergence date estimation for the branch between *Pthirus *and *Pediculus*, which the 'recent host switch' model would predict to be 7 MYA. Our estimates were much older (Table 1), and shortening the branch length artificially between *Pthirus *and *Pediculus *to resemble a divergence near 7 MYA can be easily rejected (*p *< 0.01). These analyses support an ancient duplication at that node, consistent with the 'pair of lice lost' model. Therefore, contrary to the results of the reconciliation analysis, the divergence date estimates predict a much less parsimonious explanation of current primate louse distributions: a combination of the 'recent host switch' and 'pair of lice lost' hypotheses.

**Figure 4:**
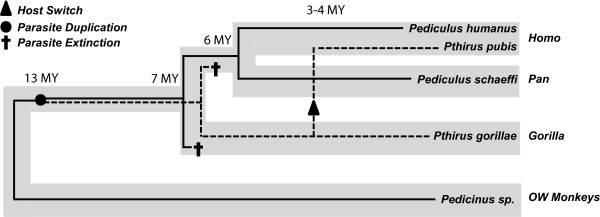
**Coevolutionary reconstruction of primate lice and their hosts based on reconciliation analysis and divergence date estimation**. Thick grey lines represent the host phylogeny for humans, chimpanzees, gorillas, and Old World monkeys. Thin black lines (solid and dashed) represent the louse lineages. This evolutionary scenario depicts a parasite duplication ca. 13 MYA leading to the extant genera *Pediculus *(solid lines) and *Pthirus *(dashed lines). One species from each lineage is depicted as having gone extinct (dagger), and a single host switch ca. 3–4 MYA is shown by an arrow within the *Pthirus *lineage. The divergence of the chimpanzee and human lice (*Pediculus spp*.) are shown as having diverged in tandem with their hosts.

Given our estimates of divergence dates, the most likely evolutionary history is that *Pthirus *and *Pediculus *diverged on an ancestor of chimpanzee, human, and gorilla roughly 13 MYA (a duplication event), with each genus then having the potential to cospeciate with descendent hosts (Figure 4). However, only the gorillas retained *Pthirus *with an extinction of *Pthirus *on the branch leading to both humans and chimpanzees (Figure 4). *Pediculus *was maintained on the lineage leading to humans and chimpanzees but lost from the gorilla lineage, and the two resulting species (*Pediculus schaeffi *and *Pediculus humanus*) diverged in tandem with their primate hosts roughly six million years ago (Figure 4). Approximately 3–4 MYA, a *Pthirus *species switched from the gorilla lineage to the lineage leading to modern humans. It is important to note that this happened after the divergence of chimpanzees and humans and that these data suggest humans acquired their pubic louse from gorillas not recently, but rather 3–4 million years ago. In total, this coevolutionary scenario requires four evolutionary steps (one duplication, two losses, and one host switch), and is a combination of both the 'recent host switch' and the 'pair of lice lost' hypotheses.

## Conclusion

Evidence suggests that *Pthirus pubis *has been associated with humans for several million years, and likely arrived on humans via a host switch from gorillas. Despite the fact that human pubic lice are primarily transmitted via sexual contact, such contact is not required to explain the host switch. Parasites often switch from a given species to a predator of that species [17], and are sometimes found to switch to unrelated hosts in communally used areas, such as roosting or nesting sites [18]. The host switch in question could have resulted from any form of contact between archaic humans and gorillas including, but not limited to, feeding on or living among gorillas. Regardless of how the transfer occurred, suitable habitat had to be available on the new human host for the host switch to be successful. For example, it is possible that the switch of *Pthirus *from gorillas to humans coincides with a change in available niche space in humans, such as the loss of body hair. Further study, however, is required to test such a hypothesis.

Because *Pthirus *has been associated with humans for several million years, this taxon can be examined in the same way that *Pediculus humanus *has to study the evolutionary history of its human host [9,19,20]. *Pthirus pubis *represents an independent, ecological replicate that went through the same evolutionary history on humans as their head/body lice, and can be used to test predictions made from *Pediculus humanus*. *Pediculus humanus *shows genetic evidence of population expansion out of Africa roughly 100,000 years ago, which is concordant with host evolutionary history [9]. However, in contrast to the shallow mitochondrial DNA (mtDNA) gene history of humans (human mtDNA coalesce to a common ancestor within 200,000 years, [21-23]), *Pediculus humanus *has three deeply divergent mtDNA lineages that share a MRCA ca. 2 million years ago, which is far older than the age of their modern human hosts [9]. Perhaps a worldwide sample of *Pthirus pubis *will mirror that of *Pediculus humanus*, and show both the population expansion 100,000 years ago and the three deeply divergent mtDNA lineages. Understanding human evolutionary history from the perspective of its parasites may provide useful insight into a brief period of history that is not fully recorded in the host fossil record or in host DNA [24]. However, if parasites are to provide much clarity, it will likely be only after many human parasites have been examined.

The advent of parsimony-based reconciliation analysis has permitted many researchers to assess phylogenetic congruence in a wide array of host-parasite assemblages. However, this method is more limited than Bayesian approaches [25] to studying cophylogenetics, which evaluate not only topological congruence but also the comparative timing of host and parasite divergences. It is imperative that we continue to put into practice the theoretical work that has propelled systematists forward in recent years. Only then can we hope to uncover the more complex interactions between hosts and parasites.

## Methods

### Specimen collection and preparation

Samples of *Pthirus gorillae *(from gorillas), *Pthirus pubis *(from humans), *Pediculus humanus *(from humans), *Pediculus schaeffi *(from chimpanzees), *Pedicinus hamadryas *(from baboons), *Pedicinus badii *(from red colobus monkeys), and one outgroup species (*Fahrenholzia reducta*) were collected for this study (Table 2). Lice in the genus *Pedicinus *parasitize only Cercopithecoid monkeys (Old World Monkeys; OWM) whereas the genera *Pediculus *and *Pthirus *parasitize only the Anthropoid primates (apes). All lice were preserved in 95% EtOH and stored at -80°C. DNA was extracted from louse specimens using the technique of Johnson and Clayton [26] and Reed *et al*. [9], which enabled extraction of whole genomic DNA from each louse while retaining the entire louse body as a voucher specimen. The Qiagen DNeasy Tissue Kit (QIAGEN Inc., Valencia, California) was used to isolate genomic DNA from the body of each louse according to louse-specific protocols [9,26,27]. After DNA extraction, lice were mounted on slides and retained as vouchers. Voucher specimens will be deposited in the Price Institute for Phthirapteran Research collection (University of Utah).

### PCR and sequencing

PCR amplification and sequencing of a portion of the mitochondrial cytochrome *c *oxidase subunit I gene (Cox1; 858 bp) was performed using the primers LCO1718 [9] and H7005 [16]. PCR amplification and sequencing of 345 bp of the nuclear elongation factor 1 alpha (EF-1α) gene were performed using the primers For3 and Cho10 [28]. Double-stranded PCR amplifications for both Cox1 and EF-1α were performed in 25 μl reaction volumes using 10 μl of Eppendorf HotMaster PCR Mix (Fisher Scientific), 1 μl of each primer (at 10 mM), and 2 μl of DNA template. The amplification protocol required an initial denaturation step of 94°C for 10 min, followed by 5 cycles of 94°C (1 min), 48°C (1 min), and 65°C (2 min), then 30 cycles of 94°C (1 min), 52°C (1 min), and 65°C (2 min) and a final extension of 65°C for 10 minutes. Amplified fragments were purified using ExoSAP-IT (USB Corporation) and sequenced in both directions. Sequences were edited using Sequencher v. 4.2.2 (Gene Codes Corporation, Ann Arbor, Michigan) and aligned by eye using Se-Al v2.0a11 . Primer sequences were removed and sequences were trimmed in reference to the translated protein sequence using Se-AL v2.01a11 and MacClade 4.0 [29]. All sequences were submitted to [Genbank: EF152552-EF152564] and alignments to [TreeBase: #SN3269]. Sequences of the Cox1 gene from the primate host taxa were downloaded [Genbank: NC001807, NC001643, NC001645, NC001992, NC008219]. EF-1α sequences were not available for several primate taxa, and were therefore not examined.

### Phylogenetic and cophylogenetic analyses

The partition homogeneity test [30] in PAUP*4.0b10 [31] was used to evaluate phylogenetic congruence of the louse Cox1 and EF-1α data sets. One thousand partition replicates were analyzed by maximum parsimony (heuristic search option with random addition replicates and tree bisection-reconnection branch swapping). Modeltest [32] was used to determine the best-fit ML model for the molecular data. Phylogenetic analyses were conducted on host and parasite data sets using maximum likelihood (ML) with branch and bound searches using the best-fit model in PAUP* 4.0b10 [31]. Nonparametric bootstraps (100 replicates) were performed to assess nodal support for the louse phylogeny. ML searches were performed with and without the 'enforce clock' constraint in order to test the hypothesis of a molecular clock in the Cox1 and EF-1α louse datasets. The resulting ML host and parasite trees with branch lengths estimated from the best-fit ML model were then used in cophylogenetic analyses.

TreeMap (v. 2.0.2; [7]) was used to determine whether host and parasite trees were more similar to one another than would be expected by chance. Default costs for evolutionary events (codivergence = 0, host switching = 1, duplication = 1, and loss = 1) were used. Significance values were calculated from a sample of 1,000 randomly generated trees.

### Divergence date estimation

Because the lice and their primate hosts showed significant codivergence and because molecular data did not differ significantly from clocklike behavior, divergence dates were estimated using methods that both adhere to and relax the molecular clock. Divergence dates were estimated in the program r8s [33], using the Langley and Fitch (LF) model which assumes a molecular clock. Dates were also estimated using a parametric Bayesian approach [34] in the program *multidivtime*. This method relaxes the molecular clock and allows rate variation among genes and lineages, and it is therefore appropriate for datasets that utilize more than one molecular marker. The topology resulting from ML analysis of the combined 2-gene data set was used in *multidivtime*. The Cox1 best-fit ML tree and the combined 2-gene topologies were used in r8s. Divergence dates were estimated using each individual gene (with branch lengths optimized on the best ML tree) as well as a combined 2-gene dataset.

For the parametric Bayesian analysis, model parameters for the F84+Γ model were estimated for each gene separately using the *baseml *program in PAML v3.14 [35]. These parameters were then used in the program *estbranches *[34,36] to estimate the ML and the variance-covariance matrix of the branch length estimates for each gene. Lastly, the program *multidivtime *[34,36], utilizing the output files from *estbranches *and implementing Markov chain Monte Carlo (MCMC) sampling, was used to estimate prior and posterior distribution of rates and divergence time estimates among lineages. The prior assumption for the mean and standard deviation of the time of the ingroup root node (rttm) was set to 3.0 time units, where 1 time unit represents 10 million years. This value corresponds to the upper limit of the split between hominoid and cercopithecoid primates. The mean and standard deviation for the prior distribution of the rate of evolution at the ingroup node (rtrate and rtratesd) was determined following the protocol of Jansa *et al*. (rttm; [37]). To avoid violation of the definition of the prior, rtratesd was set to its maximum value (equal to rtrate). The Markov chain was initialized by randomly selecting the initial parameter value and each Markov chain was sampled every 100 cycles for 1,000,000 generations with a burn in of 100,000 cycles.

A calibration point of 22.5 ± 2.5 MYA was used for the split between *Pedicinus *and *Pediculus*+*Pthirus*. This divergence of 20–25 MYA corresponds to the split between OWM and apes [38-41]. Since lice and their primate hosts show significant cospeciation, we can use this well-established host calibration based on fossil data to calibrate the louse phylogenetic trees. It is preferable to use more than one calibration point when estimating divergence dates [42,43], however the small number of nodes in our trees make that impossible. Furthermore, Reed *et al*. [9] showed that the calibration point of 20–25 MYA yielded estimated clade ages that were very similar to those estimated from a calibration of 5–7 million years between the human and chimpanzee lice (*P. humanus *and *P. schaeffi*, respectively).

## Authors' contributions

JEL and JMA collected specimens. JEL, JMA and JJK performed molecular lab work. DLR and JEL analyzed data and wrote the manuscript. All authors provided comments on initial and final drafts of the manuscript.

## Figures and Tables

**Table 1:** Divergence date estimates.

**MRCA (clade number)**	**Cox1 and EF1α**	**Cox1**	**EF1α**	**Cox1 and EF1α (r8s, 20 MYA)**	**Cox1 and EF1α (r8s, 25 MYA)**	**Cox1 (r8s, 20 MYA)**	**Cox1 (r8s, 25 MYA)**
*Pedicinus *(3)	10.63 (7.08–14.94)	10.23 (6.56–15.17)	10.91 (3.02–18.87)	11.52	13.21	11.53	14.40
*Pthirus *(1)	3.32 (1.84–5.61)	3.86 (2.05–7.49)	1.76 (0.05–6.75)	3.56	4.45	3.48	4.35
*P. schaeffi *and *P. humanus *(2)	6.39 (3.94–9.96)	6.87 (4.07–11.65)	6.65 (1.72–14.70)	5.03	6.28	4.92	6.15
*Pediculus *and *Pthirus *(4)	12.95 (9.42–17.38)	13.03 (9.25–18.21)	14.66 (7.26–22.23)	10.56	14.41	10.45	13.06
OWM/Ape Calibration	22.50 (20.13–24.87)	22.39 (20.12–24.84)	22.48 (20.12–24.87)	20.00	25.00	20.00	25.00

Mean divergence date estimates (in millions of years) for the clades shown in Figure 3 (see Figure 3 for clade numbers). Divergence estimates using a 20–25 calibration point for the split between Old World primate lice (*Pedicinus*) and Anthropoid primate lice (*Pediculus *and *Pthirus*) in *multidivtime *are given in the first three columns (95% credibility intervals in parentheses). Mean divergence date estimates using 20 MYA and 25 MYA calibration points for the split between Old World and Anthropoid primate lice using the Langley Fitch model in r8s are indicated in the final four columns for the Cox1+EF1α combined analysis and the Cox1 gene alone.

**Table 2:** Specimens examined. Louse taxa included in phylogenetic and cophylogenetic analyses.

				Genbank Accession Numbers	
Louse Species	Host Species Voucher ID	Collection Locality	Host Identification	Cox1	EF1
*Pediculus humanus*	Pdcap9.20.05.25	USA, Florida, West Palm Beach	*Homo sapiens *(WP007)	EF152552	EF152558
*Pediculus schaeffi*	Pdsch5.23.05	Uganda	*Pan troglodytes*	EF152553	EF152559
*Pthirus pubis*	Ptpub1.19.06.3	UK, Scotland, Glasgow	*Homo sapiens *(GLA 140)	EF152554	EF152560
*Pthirus gorillae*	Ptgor8.1.06.6	Uganda	*Gorilla gorilla *(051122CAWBB001)	EF152555	EF152561
*Pedicinus hamadryas*	Qnham2.4.01.2	Captive (SW Found for Biomed. Res.)	*Papio hamadryas*	AY696007	EF152562
*Pedicinus badii*	Qnbad7.24.06.9	Uganda	*Procolobus badii*	EF152556	EF152563
*Fahrenholzia pinnata*	Fzpin163	USA, Nevada, Tonopah	*Perognathus longimembris *(MLZ 2039)	EF152557	EF152564

Abbreviations are as follows: Moore Laboratory of Zoology (MLZ), Page Lab, University of Glasgow (GLA), Lice Solutions, West Palm (WP), and Maryland Gorilla Veterinary Project (MGVP).

## References

[B1] Marshall AG (1981). The Ecology of Ectoparasitic Insects.

[B2] Murray MD, Nicholls DG (1965). Studies on the ectoparasites of seals and penguins: I. The ecology of the louse *Lepidophthirus macrorhini *Enderlein on the southern elephant seal, *Mirounga leonina *(L.). Aus J Zoolog.

[B3] Kim KC, Pratt HD, Stojanovich CJ (1986). The Sucking Lice of North America.

[B4] Brooks DR (1979). Testing the context and extent of host-parasite coevolution. Syst Zoolog.

[B5] Clayton DH, Al-Tamimi S, Johnson KP, Page R (2003). The ecological basis of coevolutionary history. Tangled Trees: Phylogeny, Cospeciation and Coevolution.

[B6] Paterson AM, Gray RD, Clayton DH, Moore J (1997). Host-parasite cospeciation, host switching and missing the boat. Host-Parasite Evolution: General Principles and Avian Models.

[B7] Charleston MA, Page RDM (2002). TreeMap. v. 2.0.2. Software distributed by authors.

[B8] Page RDM, (ed.) (2003). Tangled Trees: Phylogenies, Cospeciation, and Coevolution.

[B9] Reed DL, Smith VS, Hammond SL, Rogers AR, Clayton DH (2004). Genetic analysis of lice supports direct contact between modern and archaic humans. PLoS Biol.

[B10] Stauffer RL, Walker A, Ryder OA, Lyons-Weiler M, Hedges SB (2001). Human and ape molecular clocks and constraints on paleontological hypotheses. J Hered.

[B11] Barker SC, Close RL (1990). Zoogeography and Host associations of the *Heterodoxus octoseriatus *group and *H. ampullatus *(Phthiraptera: Boopiidae) from rock wallabies (Marsupialia: *Petrogale*). Int J Parasitol.

[B12] Weckstein JD (2004). Biogeography explains cophylogenetic patterns in toucan chewing lice. Syst Biol.

[B13] Johnson KP, Adams RJ, Clayton DH (2002). The phylogeny of the louse genus *Brueelia *does not reflect host phylogeny. Biol J Linnean Soc.

[B14] Clayton DH, Price RD (1999). Taxonomy of New World Columbicola (Phthiraptera: Philopteridae) from the Columbiformes (Aves), with descriptions of five new species. Ann Ent Soc Am.

[B15] Percy DM, Page RDM, Cronk QCB (2004). Plant-insect interactions: double-dating associated insect and plant lineages reveals asynchronous radiations. Sys Biol.

[B16] Hafner MS, Sudman PD, Villablanca FX, Spradling TA, Demastes JW, Nadler SA (1994). Disparate rates of molecular evolution in cospeciating hosts and parasites. Science.

[B17] Whiteman NK, Santiago-Alarcon D, Johnson KP, Parker PG (2004). Differences in straggling rates between two genera of dove lice (Insecta: Phthiraptera) reinforce population genetic and cophylogenetic patterns. Int J Parasitol.

[B18] Johnson KP, Weckstein JD, Witt CC, Faucett RC, Moyle RG (2002). The perils of using host relationships in parasite taxonomy: phylogeny of the *Degeeriella *complex. Mol Phylog Evol.

[B19] Kittler R, Kayser M, Stoneking M (2003). Molecular evolution of *Pediculus humanus *and the origin of clothing. Curr Biol.

[B20] Ashford RW (2000). Parasites as indicators of human biology and evolution. J Med Microb.

[B21] Cann RL, Stoneking M, Wilson AC (1987). Mitochondrial DNA and human evolution. Nature.

[B22] Vigilant L, Stoneking M, Harpending H, Hawkes K, Wilson AC (1991). African populations and the evolution of human mitochondrial DNA. Science.

[B23] Ingman M, Kaessmann H, Paabo S, Gyllensten U (2000). Mitochondrial genome variation and the origin of modern humans. Nature.

[B24] Whiteman NK, Parker PG (2005). Using parasites to infer host population history: a new rationale for parasite conservation. Anim Conserv.

[B25] Huelsenbeck JP, Rannala B, Larget B (2000). A Bayesian framework for the analysis of cospeciation. Evolution.

[B26] Johnson KP, Clayton DH, Page R (2003). Coevolutionary history of ecological replicates: comparing phylogenies of wing and body lice to columbiform hosts. Tangled Trees: Phylogeny, Cospeciation and Coevolution.

[B27] Cruickshank RH, Johnson KP, Smith VS, Adams RJ, Clayton DH, Page R (2001). Phylogenetic analysis of partial sequences of elongation factor 1-alpha identifies major groups of lice (Insecta: Phthiraptera). Mol Phylog Evol.

[B28] Danforth BN, Ji S (1998). Elongation factor-1α occurs as two copies in bees: implications for phylogenetic analysis of EF1α sequences in insects. Mol Biol Evol.

[B29] Maddison WP, Maddison DR (2002). MacClade: Analysis of phylogeny and character evolution. v. 4.05.

[B30] Farris JS, Kallersjo M, Kluge AG, Bult C (1994). Testing significance of congruence. Cladistics.

[B31] Swofford DL (2002). PAUP*. Phylogenetic Analysis Using Parsimony (*and Other Methods). v. 4b10.

[B32] Posada D, Crandall KA (1998). MODELTEST: Testing the model of DNA substitution. Bioinformatics.

[B33] Sanderson MJ (2003). r8s: inferring absolute rates of molecular evolution and divergence times in the absence of a molecular clock. Bioinformatics.

[B34] Thorne JL, Kishino H (2002). Divergence time and evolutionary rate estimation with multilocus data. Systc Biol.

[B35] Yang ZH (1997). PAML: a program package for phylogenetic analysis by maximum likelihood. Comp App Biosc.

[B36] Kishino H, Thorne JL, Bruno WJ (2001). Performance of a divergence time estimation method under a probabilistic model of rate evolution. Mol Biol Evol.

[B37] Jansa SA, Barker FK, Heaney LR (2006). The pattern and timing of diversification of Philippine endemic rodents: Evidence from mitochondrial and nuclear gene sequences. Syst Biol.

[B38] Young NM, MacLatchy L (2004). The phylogenetic position of *Morotopithecus*. J Hum Evo.

[B39] Maclatchy L (2004). The oldest ape. Evol Anthropo.

[B40] Steiper ME, Young NM, Sukarna TY (2004). Genomic data support the hominoid slowdown and an Early Oligocene estimate for the hominoid-cercopithecoid divergence. Proc Natl Acad Sci USA.

[B41] Kumar S, Filipski A, Swarna V, Walker A, Hedges SB (2005). Placing confidence limits on the molecular age of the human-chimpanzee divergence. Proc Natl Acad Sci USA.

[B42] Porter ML, Perez-Losada M, Crandall KA (2005). Model-based multi-locus estimation of decapod phylogeny and divergence times. Mol Phylogen Evol.

[B43] Lee MSY (1999). Molecular clock calibrations and metazoan divergence dates. J Mol Evol.

